# Effect of metformin on the epigenetic age of peripheral blood in patients with diabetes mellitus

**DOI:** 10.3389/fgene.2022.955835

**Published:** 2022-09-26

**Authors:** Man Li, Litao Bao, Ping Zhu, Shuxia Wang

**Affiliations:** ^1^ Department of Geriatrics, The Second Medical Center and National Clinical Research Center for Geriatric Diseases, Chinese PLA General Hospital, Beijing, China; ^2^ Institute of Gerontology, Second Medical Center, PLA General Hospital, Beijing, China

**Keywords:** aging, metformin, epigenetics, DNA methylation, biomarker

## Abstract

**Background:** Metformin has been proven to have an antiaging effect. However, studies on how metformin affects global epigenetic regulation and its effect on the epigenetic clock in diabetes mellitus (DM) patients are limited. This study aims to investigate the impact of metformin on the epigenetic age in subjects with type 2 DM.

**Results:** We collected the peripheral blood of the metformin group and the no-metformin group of the 32 DM patients. Three previously established epigenetic clocks (Hannum, Horvath, and DNAmPhenoAge) were used to estimate the epigenetic age acceleration of the two groups. We defined biological age acceleration for each group by comparing the estimated biological age with the chronological age. Results were presented as follows: 1) all three epigenetic clocks were strongly correlated with chronological age. 2) We found a strong association between metformin intake and slower epigenetic aging by Horvath’s clock and Hannum’s clock.

**Conclusions:** Here, we found an association between metformin intake and slower epigenetic aging.

## Background

DNA methylation is the most widely studied epigenetic mark, and it is the process by which methyl groups are added or removed from the DNA sequence, usually at cytosine-guanine dinucleotides (CpGs). DNA methylation patterns change over the life in response to environmental factors such as diet, smoking, and stress and also with age (Rakyan et al., 2010; Bell et al., 2012; Maegawa et al., 2010). For example, while hypomethylation is common with aging, some CpG islands and gene-rich regions become hypermethylated with age (Benayoun et al., 2015). Based on age-related changes in DNA methylation, several research groups have identified what are known as DNA methylation clocks (Horvath, 2013; Nwanaji-Enwerem et al., 2018; Wagner, 2017). The epigenetic clocks have been established to be predictive of all-cause mortality (Marioni et al., 2015a;Chen et al., 2016), cancer (Kresovich et al., 2019; Yang et al., 2016), frailty, and cognitive and physical functioning (Marioni et al., 2015b). These DNA methylation clocks have been identified to predict chronological age with high accuracy and are considered as the most promising marker of aging (Lu et al., 2019). Age acceleration, a discrepancy between DNAm age and chronological age, tells us whether the person is biologically younger or older compared to his/her chronological age (Gibson et al., 2019; El Khoury et al., 2019). Accelerated epigenetic aging has been proven to be associated with many aging-related and other diseases, including cancer, Down’s syndrome, physical and cognitive decline, and all-cause mortality (Marioni et al., 2015b; Horvath et al., 2015; Marioni et al., 2015a; Daniel et al., 2018).

Metformin is a widely used medication that has been used as the first-line oral treatment for type 2 diabetes (Inzucchi, 2002). Recent advances revealed that this drug, in addition to its glucose-lowering action, might be a promising target for aging (Barzilai et al., 2016; Pernicova and Korbonits, 2014; Martin-Montalvo et al., 2013; Johnson et al., 2013; Cabreiro et al., 2013). It appears to target a number of age-related mechanisms. At a molecular level, metformin leads to the activation of adenosine-activated protein kinase (AMPK) and increases antioxidant protection (Pernicova and Korbonits, 2014; Martin-Montalvo et al., 2013). Metformin could exert the inhibition of the mammalian target of rapamycin (mTOR) signaling (Nair et al., 2014). Inhibition of this pathway extends the lifespan in model organisms and confers protection against a growing list of age-related pathologies (Johnson et al., 2013). Preclinical studies of metformin suggest that metformin robustly increases the lifespan in *C. elegans* by up to 36% (Cabreiro et al., 2013). There is a lot of evidence that metformin is a promising antiaging drug. However, studies on how metformin affects global epigenetic regulation and its effect on the epigenetic clock are limited. The aim of the study was to investigate the metformin-induced antiaging effect and its effects on the genome-wide DNA methylation in human peripheral blood. We conducted this study to investigate the pathways of metformin in real-life physiological conditions in humans. This is important given the polypotent effects of metformin, and such research could lead to new and important targets not only for the treatment of DM but also for other diseases.

## Methods

### Study population

Diabetes mellitus (DM) was defined as the presence of diabetes symptoms and a resting plasma glucose concentration ≥200 mg/dl, a fasting plasma concentration ≥126 mg/dl, a 2h plasma glucose concentration ≥200 mg/dl in a 75 g oral glucose tolerance test or use of a hypoglycemic agent or other medications for DM. The candidates were excluded if they had severe heart failure, active infectious disease, history of malignancy, or end-stage renal disease, or were in a deep coma. Patients were also excluded if they have any other chronic disease other than diabetes, such as cardiovascular disease, respiratory disease, tumor, and rheumatic immune disease, confirm that the participants did not use antibiotics, immunosuppressive medications, corticosteroids, or pharmaceutical-grade probiotics during at least 2 months leading up to blood collection. Also, confirm that no subject had diarrhea within 7 days leading into the study. All the participants reported that they were on a normal diabetic diet. These patients were divided into two groups based on whether they were on metformin medication with a stable dosage of 0.5 g/d for at least 5 years. The characteristics of the study population were summarized in Table 1. None of the two groups included in the study had any disease other than diabetes and neither group had received any other medications in the past 5 years. All participants provided written informed consent and the study was approved by the ethics board of the Chinese PLA general hospital.

**TABLE 1:** Characteristics of the study group.

Characteristics of the study group				
Characteristic	Total	No-metformin group	Metformin group	*p* value
Male	100%	100%	100%	-
Age, years, mean ± SD	73.3 ± 5.60	73.6 ± 5.90	72.9 ± 6.30	0.56
Smoking, n (%)	12(37.50)	5(31.25)	7(43.75)	0.09
BMI and mean ± SD	25.3 ± 3.40	25.2 ± 2.90	25.4 ± 3.70	0.98
Fasting plasma glucose, mmol/l, mean ± SD	6.27 ± 2.70	6.19 ± 2.08	6.31 ± 3.12	0.12
HbA1c, %	(5.35 ± 1.07)%	(5.65 ± 0.98)%	(5.24 ± 1.20)%	0.65

### Genomic DNA extraction and quality control

Fasting peripheral blood samples were collected in the morning and then stored at −80°C until use. Approximately, 500 ng of genomic DNA from each sample was used for sodium bisulfite conversion using the EZ DNA Methylation-Gold Kit (Zymo Research, United States) in accordance with the manufacturer’s instructions. DNA from a total of 32 participants was assayed using Illumina’s Infinium human methylation 850 Beadchip as previously described. Genome-wide DNA methylation was assessed using the Illumina Infinium human methylation 850K BeadChip (Illumina Inc, United States) which covers 99% of all RefSeq genes and contains 853,307 sites. The process was performed according to the manufacturer’s protocols. Quality control of methylation data including intensity readouts, filtering, and cellar composition was adapted from Hannon et al. (2016) and was done using the R package (version 4.0.0). Load EPIC’s original IDAT files and filter out probe sites according to the following principles: 1) filter out probes used to filter *p* values (≧0.01) 2). In more than 5% of samples, the probes in beads smaller than three were filtered out. 3) Filter out all non-CpG probes contained in the dataset. 4) Filter all SNP-related probes. 5) Filter all probes in chromosomes X and Y.

### Determinations of epigenetic age and epigenetic age acceleration

The array data (IDAT files) was analyzed using the ChAMP package in R for deriving the methylation level. The methylation status of all the probes was denoted as β value, which is the ratio of the methylated probe intensity to the overall probe intensity (sum of methylated and unmethylated probe intensities plus constant α, where α = 100). CpG site (probe) intensities were transformed to β values with a standard equation in which beta is the ratio of the methylated probe (m) intensities to the overall intensities (m + u+α, where α is the constant offset, 100, and u is the unmethylated probe intensity). The resulting β values ranged from 0 (completely unmethylated) to 1 (fully methylated). It is generally believed that a β value greater than 0.8 is hypermethylated, while a β value less than 0.2 is hypomethylated, and a β value in the range of 0.2–0.8 is partially methylated. The DNA methylation ages were measured using the epigenetic clock (Horvath, 2013). The epigenetic clock was defined as an age prediction method based on DNA methylation levels at 353 CpG sites. The epigenetic age acceleration value was calculated by subtracting the actual chronological age from the DNAm age (Horvath, 2013). Another measure of acceleration (acceleration residual) equaled the residual resulting from linear regressing the DNAm age on the chronological age (Horvath, 2013). Using the processed DNAm data, DNAm age was calculated using the R code provided by the clock developers (Hannum et al., 2013; Horvath, 2013; Levine et al., 2018).

### Statistical analysis

Baseline characteristics were conducted using Stata 14 software (StataCorp, Inc., College Station, TX, United States). The data are presented as averages and standard deviations unless otherwise stated. Associations between chronological age and DNAm age were analyzed using standardized regression coefficients. The Kruskal–Wallis test was performed to determine the significant difference between the metformin group and the control group. For each of the three epigenetic clocks, we defined epigenetic age acceleration by regressing the DNAm age to chronological age and calculating the difference between observed and fitted DNAm age as epigenetic age acceleration (EAA). We calibrated the fitted DNAm age for the entire analyzed sample and restricted it to a random subcohort; we did not observe meaningful differences using either method; therefore, we used the DNAm age fit to the full dataset. We estimated age acceleration with and without adjustment for blood cell composition (BCC), as it varies with age (Chen et al., 2016). Pearson correlations were used to examine DNAm age and epigenetic age acceleration metric correlations with chronological age. We adjusted for confounding by age, body mass index (BMI; kg/m2, continuous), and smoking because they may influence DNAm age. R Studio was used to perform the statistical analysis.

## Results

### Characteristics of the study participants

All subjects were male, with an average of 73.3 years. Based on the medications of metformin, the patients were divided into the no-metformin group and the metformin group. A total of 16 DM patients with at least 5-year of medications with a stable dosage of 0.5 g/d were included in the metformin group and the other 16 DM patients with no metformin medications in the past 5 years were included in the no-metformin medication group. A total of 32 patients were included in the study. The characteristics of the study population are summarized in Table 1.

### Prediction of epigenetic age using the epigenetic clock

**FIGURE 1:**
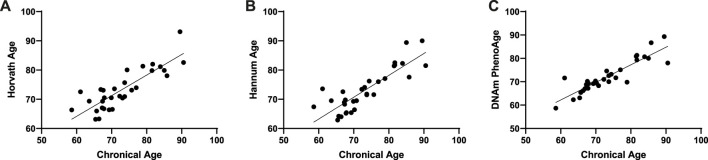
Correlation between epigenetic age and chronological age.

We used an Illumina Infinium 850k array to evaluate the effect of metformin on DNA methylation in patients. To verify the epigenetic clock predictors in our cohort, we correlated epigenetic age with chronological age, as described earlier. As expected, all three epigenetic clocks were strongly correlated with chronological age (Hannum: Pearson r = 0.88, *p* < 0.001; Horvath: Pearson r = 0.85, *p* < 0.001; DNAmPhenoAge: Pearson r = 0.83, *p* < 0.001) (Figures 1A–C).

### The association between metformin intake and slower epigenetic aging in patients with DM

**FIGURE 2:**
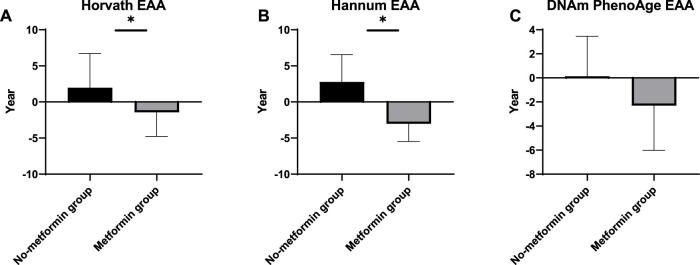
Epigenetic Age Acceleration of the two groups by the three epigenetic clocks.

We evaluate age acceleration developed by the three clocks. *t*-tests were conducted to verify whether the metformin intake was associated with slower epigenetic aging in the peripheral blood of the patients with DM. The meta-analyzed estimates for EAA, adjusted for chronological age, sex, BMI, and smoking variables, exhibited statistically significant associations for metformin intake and EAA measures with the exception of the DNAmPhenoAge clock. A positive (negative) value of accelerated age indicates that the DNAm age is older (younger) than its actual age. The Horvath EAA of the no-metformin group exceeded that of the metformin group by 2.77 years (*p* = 0.04) (Figure 2A). The Hannum EAA of the no-metformin group exceeded that of the metformin group by 3.43 years (*p* = 0.04) (Figure 2B). The DNAmPhenoAge EAA values did not show significant differences between the two groups, but these differences show a similar trend to accelerating differences (Figure 2C).

### Multivariable adjustment

**FIGURE 3:**
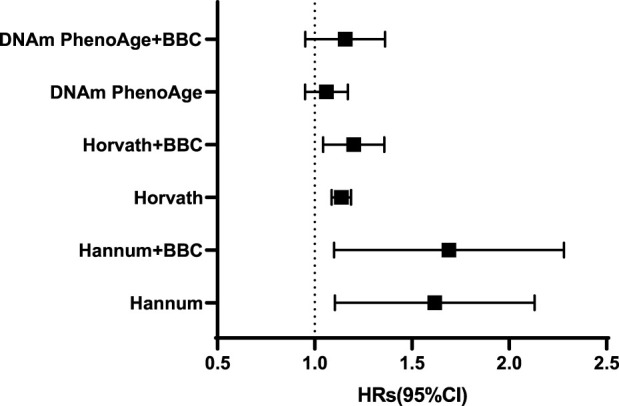
Adjusted hazard ratios [and 95% confidence intervals (CI)] for the epigenetic age acceleration.

The meta-analyzed estimates for EAA, adjusted for chronological age, sex, race, and smoking variables, exhibited statistically significant associations for metformin intake. After adjusting for other risk factors in the multivariable model, statistical significance persisted for EAA measures for two clocks: the Horvath clock (HR, 1.19 [95% CI, 1.09–1.31]; P < 0.05) and the Hannum clock (HR, 1.75 [95% CI, 1.05–2.05]; P < 0.05; Table 2). The meta-analyzed point estimate for Hannum EAA had the largest magnitude association (hazard ratio [HR], 1.75 [95% CI, 1.05–2.05]) (Figure 3). Although the meta-analyzed measures of association for EAA measures for the DNAmPhenoAge EAA were not statistically significant after multivariable adjustment, their directionality remained consistent relative to the associations observed on unadjusted analysis.

**TABLE 2:** Epigenetic age acceleration metrics and metformin intake using the full sample of DM patients.

Epigenetic clocks	HR (95%CI)	P
Hannum	1.75(1.05–2.05)	< 0.01
Hannum + BBC	1.87(1.03–2.17)	< 0.01
Horvath	1.19(1.09–1.13)	0.04
Horvath + BBC	1.23(1.03–1.34)	0.02
DNAmPhenoAge	1.06(0.95–1.17)	0.14
DNAmPhenoAge + BBC	1.21(0.93–1.22)	0.1

## Discussion

We performed a genome-wide methylation study to investigate the effect of metformin on DNA methylation using baseline blood samples. We demonstrated that metformin intake was associated with slower epigenetic aging. In the present study, three clocks were used to determine the effect of metformin on DM patients. Our research showed that Hannum EAA had the strongest association with metformin intake. Therefore, our study strengthens the view reported earlier that the epigenetic clock is a relatively accurate reflection of a person’s biological age.

### Metformin and aging

Metformin is an approved drug to treat diabetes, but it also seems to target some of the mechanisms associated with aging. Particularly for aging, metformin can lead to decreased insulin levels, a decreased IGF-1 signaling pathway (Yang et al., 2020; Admasu et al., 2018), and mTOR inhibition (Vazirpanah et al., 2019; Howell et al., 2017), inhibition of mitochondrial complex 1 in the electron transport chain and reduction of endogenous reactive oxygen species (ROS) production (Nguyen et al., 2019; Bridges et al., 2014), activation of AMPK (Howell et al., 2017; Duca et al., 2015), and a decrease of DNA damage (Zhang et al., 2016; Na et al., 2013). It has been reported that metformin increases the lifespan of *C. elegans* by altering microbial folate and methionine metabolism (Cabreiro et al., 2013). It also improves the health span and lifespan of mice and humans (Martin-Montalvo et al., 2013; Forslund et al., 2015). Moreover, metformin has been proven to interact with several known longevity pathways such as dietary restriction (DR) (Dhahbi et al., 2005; Madeo et al., 2019).

### The epigenetic clock

Recent evidence suggests that the epigenetic clock is the most promising marker of aging. The epigenetic clock has been reported to track biological aging associated with morbidity and mortality. The result of a meta-analysis including 13,089 participants showed that epigenetic clocks can predict all-cause mortality (Chen et al., 2016). Epigenetics clock is considered to be the most promising biomarker of biological age when compared with telomere length and other biomarkers (Jylhävä et al., 2017). Similarly, by comparing different estimation methods, the researchers identified DNA methylation as the most promising biomarker for predicting age (Lee et al., 2016). Therefore, many antiaging measures use epigenetic age to evaluate the effectiveness of interventions (Fahy et al., 2019; Sae-Lee et al., 2018). Metformin has long been considered as an “anti-aging” drug, based on preclinical experiments with lower-order organisms and numerous retrospective data (Barzilai et al., 2016). However, the molecular mechanisms remained unclear and the underlying mechanisms need to be better understood. Previous studies have reported the effect of metformin on epigenetics (Banerjee et al., 2016; Zhong et al., 2017; García-Calzón et al., 2017). In a small sample size study (*n* = 12), Elbere et al. (2018) showed an altered blood DNA methylation profile following the use of metformin in nondiabetic participants. However, studies focused on metformin’s effect on the epigenetic clock are limited. The analysis of epigenetic differences between elderly diabetic patients with and without metformin is helpful to find possible intervention targets.

### The underlying mechanisms: metformin and AMPK

Our study confirms that epigenetic ages are younger in DM patients with medication of metformin. AMPK plays a major regulatory role in cell energy homeostasis by directly phosphorylating metabolic enzymes and nutrient transporters, and indirectly promoting mitochondrial biogenesis and the deactivation of nuclear genes in functional mitochondrial biogenesis. AMPK as a target for promoting healthy aging is associated with its role in multiple signaling pathways. 1) TOR pathway: downregulation of the TOR pathway extends the lifespan in *C. elegans*, fruit flies, and mice (Johnson et al., 2013). The prevailing view of AMPK/TOR interaction is that AMPK is primarily an upstream inhibitor of TOR (Inoki et al., 2003; Gwinn et al., 2008). 2) FOXOs pathway: rIIS is the most powerful and least controversial candidate to promote healthy aging, rIIS dramatically increases life expectancy and extends healthy aging in a variety of organisms, including mammals. The only member of the FOXO transcription factor family that is activated by rIIS completely requires rIIS-mediated longevity. In *C. elegans*, AMPK might activate FOXO, thus performing the antiaging effect. 3) Sirtuins pathway: SIRT1 gene plays an antiaging role by improving efficiency in inducing and maintaining pluripotent states. AMPK can activate SIRT1 by changing the nicotinamide adenine dinucleotide: reduced nicotinamide adenine dinucleotide (NAD: NADH) ratio. To sum up, AMPK influences the aging process through a variety of pathways (Ong and Ramasamy, 2018).

It has been reported that AMPK activator metformin leads to increased trimethylation of H3K79 and regulates mitochondrial biogenesis and senescence through H3K79 methylation (Karnewar et al., 2018). AMPK-mediated phosphorylation resulted in the activation of histone acetyltransferase 1(HAT1) (Marin et al., 2017). H3K4me3 antagonizes the HIR/Asf1/Rtt106 repressor complex to promote histone gene expression and extend chronological lifespan. New research indicated that changes in AMPK phosphorylation following the changes in histone 3 (H3K9) acetylation and methylation status (Dziewulska et al., 2020).

### Limitations

Here, we found an association between metformin intake and slower epigenetic aging of the DM patients, and metformin is a potentially promising antiaging drug; there are still some limitations to our study. The sample size is relatively small and limited to a single center. There are still some lifestyle factors that can influence DNA methylation, such as any potential difference between diet and physical activity levels among the groups. Although all of the participants reported that they were on a normal diabetic diet, we know that the Chinese diet is complex and these factors cannot be compared in most cases because we try to avoid the effects of diet on DNA methylation levels.

## Conclusion

We found an association between metformin intake and slower epigenetic aging in DM patients.

## Data Availability

The datasets presented in this study can be found in online repositories. The names of the repository/repositories and accession number(s) can be found in the article/Supplementary Material.

## Abbreviations

AMPK, adenosine activated protein kinase; DM, diabetes mellitus; DMPs, differential methylation positions; mTOR, mammalian target of rapamycin; BMI, body mass index; BCC, blood cell composition; EAA, epigenetic age acceleration; CpGs, cytosine-guanine dinucleotides; NAD, nicotinamide adenine dinucleotide; NADH, reduced nicotinamide adenine dinucleotide.
